# Loss of the Nuclear Protein RTF2 Enhances Influenza Virus Replication

**DOI:** 10.1128/JVI.00319-20

**Published:** 2020-10-27

**Authors:** Bing Shao Chia, Bo Li, Ang Cui, Thomas Eisenhaure, Raktima Raychowdhury, David Lieb, Nir Hacohen

**Affiliations:** a Harvard University Virology Program, Harvard Medical School, Boston, Massachusetts, USA; b Broad Institute of MIT and Harvard, Cambridge, Massachusetts, USA; c Center for Cancer Research, Massachusetts General Hospital, Harvard Medical School, Boston, Massachusetts, USA; Hudson Institute of Medical Research

**Keywords:** RTF2, antiviral, influenza virus, innate immunity, interferon, interferon-stimulated genes, restriction factor, transcription

## Abstract

While hundreds of genes are induced by type I interferons, their roles in restricting the influenza virus life cycle remain mostly unknown. Using a loss-of-function CRISPR screen in cells prestimulated with interferon beta (IFN-β), we identified a small number of factors required for restricting influenza A virus replication. In addition to known components of the interferon signaling pathway, we found that replication termination factor 2 (RTF2) restricts influenza virus at the nuclear stage (and perhaps other stages) of the viral life cycle, based on several lines of evidence. First, a deficiency in RTF2 leads to higher levels of viral primary transcription, even in the presence of cycloheximide to block genome replication and secondary transcription. Second, cells that lack RTF2 have enhanced activity of a viral reporter that depends solely on four viral proteins that carry out replication and transcription in the nucleus. Third, when the RTF2 protein is mislocalized outside the nucleus, it is not able to restrict replication. Finally, the absence of RTF2 leads not only to enhanced viral transcription but also to reduced expression of antiviral factors in response to interferon. RTF2 thus inhibits primary influenza virus transcription, likely acts in the nucleus, and contributes to the upregulation of antiviral effectors in response to type I interferons.

**IMPORTANCE** Viral infection triggers the secretion of type I interferons, which in turn induce the expression of hundreds of antiviral genes. However, the roles of these induced genes in controlling viral infections remain largely unknown, limiting our ability to develop host-based antiviral therapeutics against pathogenic viruses, such as influenza virus. Here, we performed a loss-of-function genetic CRISPR screen in cells prestimulated with type I interferon to identify antiviral genes that restrict influenza A virus replication. Besides finding key components of the interferon signaling pathway, we discovered a new restriction factor, RTF2, which acts in the nucleus, restricts influenza virus transcription, and contributes to the interferon-induced upregulation of known restriction factors. Our work contributes to the field of antiviral immunology by discovering and characterizing a novel restriction factor of influenza virus and may ultimately be useful for understanding how to control a virus that causes significant morbidity and mortality worldwide.

## Abstract (precis)

Viral infection triggers the secretion of type I interferons, which in turn induce the expression of hundreds of antiviral genes. However, the roles of these induced genes in controlling viral infections remain largely unknown, limiting our ability to develop host-based antiviral therapeutics against pathogenic viruses, such as influenza virus. Here, we performed a loss-of-function genetic CRISPR screen in cells prestimulated with type I interferon to identify antiviral genes that restrict influenza A virus replication. Besides finding key components of the interferon signaling pathway, we discovered a new restriction factor, RTF2, which acts in the nucleus, restricts influenza virus transcription, and contributes to the interferon-induced upregulation of known restriction factors. Our work contributes to the field of antiviral immunology by discovering and characterizing a novel restriction factor of influenza virus and may ultimately be useful for understanding how to control a virus that causes significant morbidity and mortality worldwide.

## INTRODUCTION

The innate immune response provides an essential first line of defense against invading pathogens. Although type I interferons (IFN) were discovered approximately 6 decades ago (1) and hundreds of interferon-stimulated genes (ISGs) have subsequently been discovered via gene expression profiling studies (2, 3), only a small number have been characterized in depth (e.g., IFITM3 [4–6], Mx1 [7], protein kinase R [PKR] [8–10], OAS-RNase L [11–14]). Most of these ISGs remain relatively uncharacterized, especially in terms of their antiviral effect sizes, their targets, and their mechanisms of viral restriction (15). A deeper understanding of how virus restriction occurs naturally may inform novel therapeutic strategies against viruses that pose major health threats, such as influenza virus, which causes an estimated 3 million to 5 million cases of severe illness and 0.29 million to 0.65 million deaths worldwide annually (16, 17).

This knowledge gap has been addressed in part by studies that systematically overexpressed each of 400 ISGs to determine their effects on virus replication, identifying dozens of factors with antiviral activity (15, 18). However, this does not address the question of whether the individual ISGs are required, rather than sufficient, for restricting virus replication. Although loss-of-function screens have been done before, very few ISGs have been identified through such an approach (4, 19). For instance, in the genome-wide small interfering RNA screen performed by Brass et al. to identify host factors that are involved in influenza A virus (IAV) infection, only 4 genes were reported to increase IAV replication and/or gene expression (4).

We hypothesized that a reason for not identifying more anti-IAV factors through such genome-wide screens is because the screens had not been designed and optimized specifically for such a purpose. Furthermore, the previously published IAV-specific screens were not performed in the presence of IFN treatment, and hence, it is possible that only restriction factors that have constitutively high expression levels and/or that exhibit very potent restriction effects would be identifiable. We thus hypothesized that if we were to tailor our screen conditions specifically to identify antiviral factors, such as pretreating cells with IFN prior to IAV infection, we could potentially identify novel host factors that restrict IAV infection in a systematic and unbiased way. Such a genome-wide screen not only could potentially identify novel antiviral effectors but also could identify cofactors or downstream effectors that are necessary for previously reported ISGs to function (such as ZMPSTE24, which is required for IFITM3 activity but which is not upregulated by IFN [20]).

In our study, we screened for factors that restrict IAV replication in response to interferon beta (IFN-β) exposure in A549 lung epithelial cells and present evidence that replication termination factor 2 (RTF2) restricts influenza virus replication in the nucleus and affects the response to type I interferons.

## RESULTS

### A genome-wide CRISPR screen for factors that restrict influenza virus reveals components of the type I interferon pathway but not individual interferon-stimulated effector genes.

**FIG 1:**
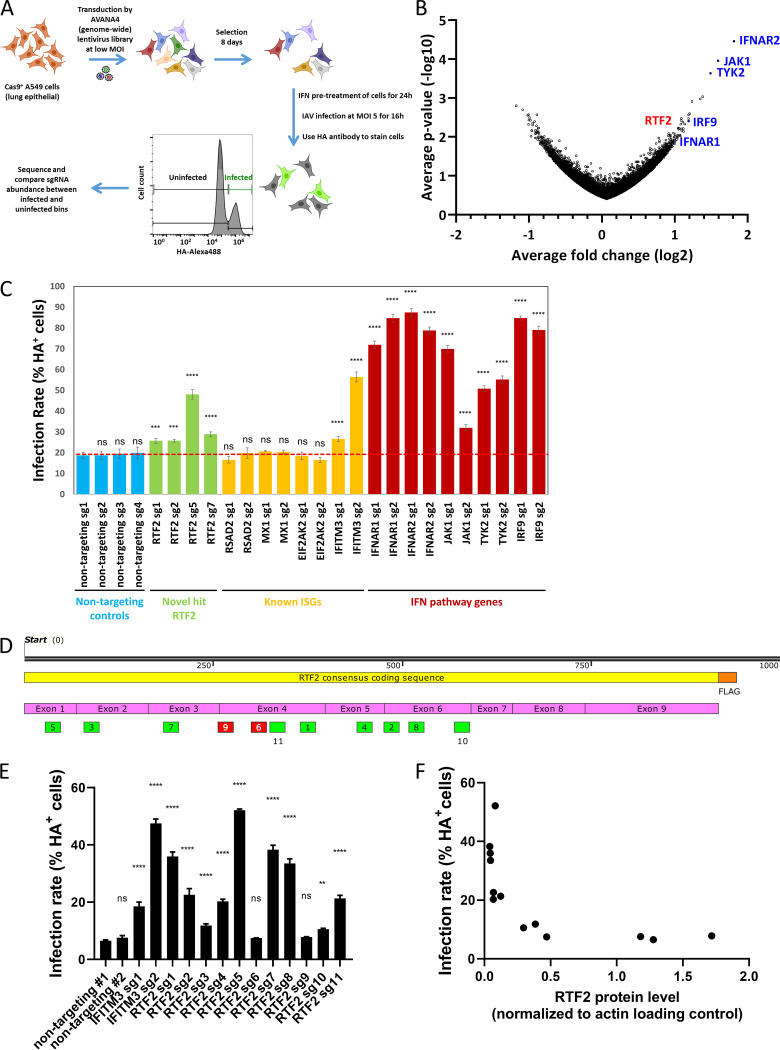
Screen identifies RTF2 as a potential antiviral candidate gene. (A) Schematic overview of the CRISPR screen for sgRNAs that permit infection in the presence of type I interferon. (B) Volcano plot showing enrichment of sgRNAs in infected cells versus uninfected cells based on the fold change in expression (*x* axis) and statistical significance (*y* axis). Blue text shows known genes of the IFN pathway. (C) IAV infection rates based on HA surface protein levels in A549 cells transduced with single sgRNAs and Cas9 lentiviruses (in triplicate). Cells were selected with puromycin for 7 days, pretreated with 200-U/ml IFN-β for 24 h, and infected with IAV at an MOI of 5 (adjusted for cell counts) for 16 h. *P* values were determined by one-way ANOVA and Dunnett’s multiple-comparison test against nontargeting sgRNA 1. ****, *P* ≤ 0.0001; ***, *P* ≤ 0.001; ns, not significant. (D) Schematic showing the distribution of the different RTF2 sgRNAs (boxed numbers) along the consensus coding sequence of RTF2. (E) IAV infection rates based on HA surface protein levels in A549 cells transduced with Cas9 and individual RTF2 sgRNAs. *P* values were determined by one-way ANOVA and Dunnett’s multiple-comparison test against nontargeting sgRNA 1. ****, *P* ≤ 0.0001; **, *P* ≤ 0.01; ns, not significant. (F) Scatterplot showing the relationship of infection rate (as measured by the percentage of HA-positive cells) and RTF2 expression (normalized to that for the actin loading control) in cells that were transduced with the different nontargeting and RTF2 sgRNAs shown in panel E. Spearman correlation analysis gave an *r* value of −0.85 with a *P* value of 0.0004.

To perform a genome-wide CRISPR screen, we generated A549 lung epithelial cells that stably express Cas9 (A549-Cas9 cells) and transduced these A549-Cas9 cells with the AVANA4 lentivirus library (21), which contains 74,700 single guide RNAs (sgRNAs) targeting 18,675 protein-coding genes, as well as 1,000 nontargeting sgRNAs. We added IFN-β to ∼300 million library-transduced cells and after 24 h infected the cells with the influenza A virus A/Puerto Rico/8/1934 (PR8) at a multiplicity of infection (MOI) of 5. After 16 h, influenza virus-infected cells were flow sorted based on the level of viral hemagglutinin (HA) on the cell surface, and their genomic DNA was extracted for sequencing to identify enriched or depleted sgRNAs (Fig. 1A). To find antiviral factors, we identified sgRNAs that were enriched in cells that were within the top 10% of HA-expressing cells (infected, susceptible) versus cells with median viral HA expression in the lower peak (uninfected, protected) (Fig. 1B). The top three hits were interferon alpha/beta receptor 2 (IFNAR2), nonreceptor tyrosine-protein kinase 2 (TYK2), and Janus kinase 1 (JAK1), and further down the list were interferon alpha/beta receptor 1 (IFNAR1) and interferon regulatory factor 9 (IRF9), all of which are essential components in the IFN signaling pathway (22). These were further validated using additional single guides. However, we did not recover previously reported antiviral ISGs (23), such as the genes for IFITM3 (4–6), Mx1 (7), PKR (8–10), OAS3 (13), RNase L (14), RSAD2 (viperin) (24), TRIM22 (25), and ISG15 (26). This could be due to a combination of reasons, such as each gene having a small effect size in the context of many IFN-induced antiviral genes (as well as the expected reduction in effect size due to incomplete editing [27] and potential enrichment for nonedited cells that had higher fitness), as well as an inherent limitation of the assay that we chose (only genes acting on IAV life cycle steps up to HA trafficking would be recoverable). In contrast, since the IFN pathway components found in our screen are known to be required for inducing many ISGs at once, the impact of deleting them is to undo the antiviral effects of IFN. Using a more sensitive arrayed format to validate some of our primary screen hits, we found that of four well-studied antiviral effector genes, those for IFITM3, Mx1, PKR, and RSAD2, only the gene for IFITM3 showed an increase in infection compared to the nontargeting guides (Fig. 1C), while guides targeting IFN pathway components gave the expected increase in infection.

### Validation of RTF2 as an antiviral factor.

**FIG 2:**
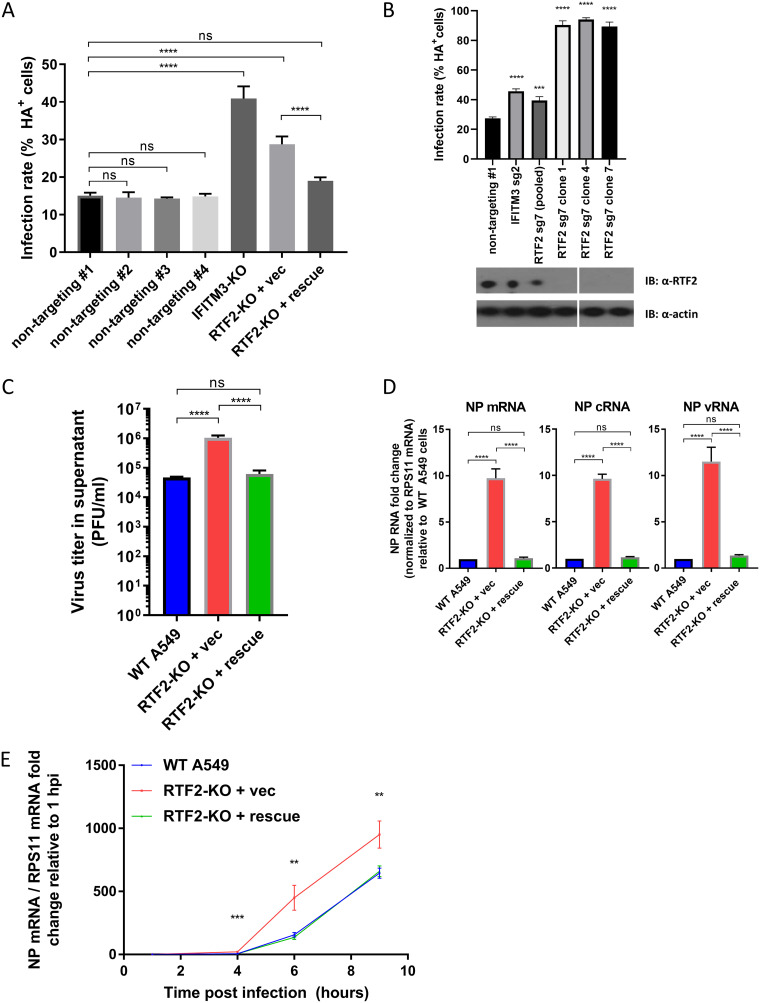
The loss of RTF2 increases the IAV infection rate in interferon-treated cells. (A) IAV infection rates based on HA surface protein levels in RTF2-KO cells and RTF2-rescued cells. RTF2-KO cells were transduced with either an empty vector or an sgRNA-resistant RTF2 cDNA to restore RTF2 expression. ****, *P* ≤ 0.0001, by one-way ANOVA and Tukey’s multiple-comparison test. (B) (Top) IAV infection rates based on HA surface protein levels in A549 cells transduced with nontargeting sgRNA, IFITM3 sgRNA, or RTF2 sgRNA. The three rightmost columns denote three separate RTF2-KO cell clones isolated and cultured from a pool of RTF2 sgRNA 7 polyclonal cells. *P* values were determined by one-way ANOVA and Dunnett’s multiple-comparison test against the nontargeting sgRNA sample. ****, *P* ≤ 0.0001; ***, *P* ≤ 0.001. (Bottom) The Western blot (immunoblot [IB]) image shows RTF2 protein expression in the isolated RTF2-KO cell clones. (C) Quantification of IAV progeny virions produced by infected WT A549, RTF2-KO, and RTF2-rescued cells. Cells were first pretreated with 200-U/ml IFN-β for 24 h, before IAV infection at an MOI of 5 for 16 h. The cell culture supernatant was collected and used to perform plaque assays to quantify the amount of infectious particles released. ****, *P* ≤ 0.0001, by one-way ANOVA and Tukey’s multiple-comparison test. (D) IAV transcription and replication for WT, RTF2-KO, and RTF2-rescued cells. Cells were pretreated with 200-U/ml IFN-β for 24 h, before IAV infection at an MOI of 5 for 9 h. Total RNA was used for strand-specific RT-PCR and qPCR to quantify the levels of viral NP mRNA, cRNA, and vRNA. ****, *P* ≤ 0.0001, by one-way ANOVA and Tukey’s multiple-comparison test. (E) Time course of IAV NP mRNA in WT, RTF2-KO, and RTF2-rescued cells. *P* values were determined by one-way ANOVA and a multiple-comparison test. ***, *P* ≤ 0.001; **, *P* ≤ 0.01. vec, vector; sg2, sgRNA 2; sg7, sgRNA 7.

Among the top hits in our primary screen, RTF2 (also known as RTFDC1) was found to be an antiviral gene, with little being known about its cellular function (28) or role in infection. As further validation, we found that independent guides, which were designed to spread out across the entire RTF2 gene (Fig. 1D), led to higher HA levels in IAV-infected IFN-β-pretreated A549 cells than in cells that received nontargeting guides (Fig. 1E), with the effect size being inversely correlated to RTF2 expression levels (Fig. 1F). In addition, overexpression of sgRNA-resistant RTF2 in RTF2-depleted cells restored protection against IAV infection (Fig. 2A). Because gene editing with CRISPR-Cas9 does not lead to a loss of function in all cells of a polyclonal population, we isolated individual clones of cells treated with a strong guide against RTF2. Indeed, the effect size of knocking out RTF2 was larger in several clonal knockout lines in which RTF2 protein expression was low or undetectable (Fig. 2B). Subsequent experiments were conducted using clone 1 cells (RTF2-knockout [RTF2-KO] cells) and clone 1 cells expressing sgRNA-resistant RTF2 (RTF2-rescued cells), as such experimental data would be more reproducible due to the homogeneous nature of clonal cells. These clonal RTF2-KO cells had no detectable RTF2 protein and had a 2-bp deletion (chromosome 20 bp 56,474,701 and 56,474,702) leading to a frameshift in one allele and a 24-bp deletion (chromosome 20 bp 56,474,701 to 56,474,724) in the other allele (located within exon 3 of the RTF2 transcript ENST00000357348.9).

Besides monitoring the infection rate by measuring the percentage of HA-positive cells, we also monitored the production of infectious particles in the IAV-infected, IFN-β-pretreated cells at 16 h postinfection, by collecting the cell culture supernatant and performing plaque assays on Vero cells. We noticed that a significantly higher virus titer was observed in RTF2-KO cells than in wild-type (WT) A549 cells and RTF2-rescued cells (Fig. 2C). Based on these data, we conclude that RTF2 has an antiviral role during IAV infection.

### Viral RNA levels are increased in RTF2-deficient cells at early time points.

To monitor viral RNA levels, we used quantitative PCR (qPCR) to quantify IAV RNAs from infected cells and found that the nucleoprotein (NP) mRNA, cRNA, and viral RNA (vRNA) levels were higher in RTF2-KO cells than in wild-type A549 cells or RTF2-rescued cells (Fig. 2D). We also tracked the kinetics of infection by monitoring NP mRNA levels over time and noticed that the difference in NP mRNA levels between RTF2-KO cells and RTF2-rescued cells could be detected even at 4 h postinfection (hpi) (Fig. 2E). Our data suggest that depletion of RTF2 leads to increased replication during an early step in the IAV life cycle.

### RTF2’s localization is important for its antiviral role.

**FIG 3:**
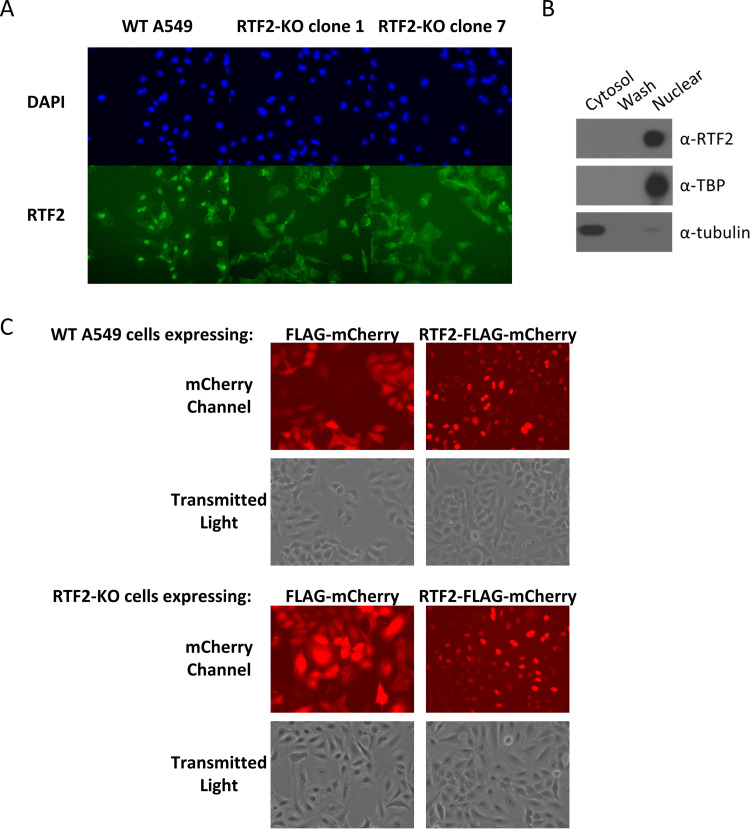
RTF2 localizes to the nucleus. (A) Immunofluorescence of WT A549 cells and two distinct clones of RTF2-KO cells. (B) Biochemical fractionation of WT A549 cells into cytosolic and nuclear fractions. Cells were first suspended in HEPES-sucrose-Ficoll (HSF) buffer containing digitonin to extract the cytosolic proteins and washed once with HSF buffer, before lysing the nuclear pellet in RIPA buffer to release the nuclear proteins. TATA-binding protein (TBP), a nuclear protein, and tubulin, a cytosolic protein, were included as controls for the fractionation protocol. (C) Live-cell imaging of cells expressing either mCherry or the RTF2-mCherry fusion protein. WT A549 (top) and RTF2-KO cells (bottom) were transduced with lentiviruses that encode either FLAG-mCherry or RTF2-FLAG-mCherry and cultured for a week, before they were visualized under a wide-field epifluorescence microscope.

We hypothesized that RTF2 functions in the nucleus, because prior work showed that RTF2 localizes to replication forks in the nucleus (28) (and has to be removed from stalled replication forks to maintain genome stability) and that the RTF2 homolog in Schizosaccharomyces pombe inhibits replication restart to achieve efficient replication termination at the site-specific replication barrier, *RTS1* (29). Through immunofluorescence staining of wild-type A549 cells and two different RTF2-KO cell clones (clones 1 and 7) with the endogenous anti-RTF2 antibody, we found that RTF2 appears to be localized mainly in the nucleus (Fig. 3A). This was corroborated by biochemical fractionation (Fig. 3B). In addition, live-cell imaging of wild-type A549 and RTF2-KO cells transduced to express mCherry-tagged RTF2 showed a clear nuclear localization of the overexpressed RTF2 (Fig. 3C).

**FIG 4:**
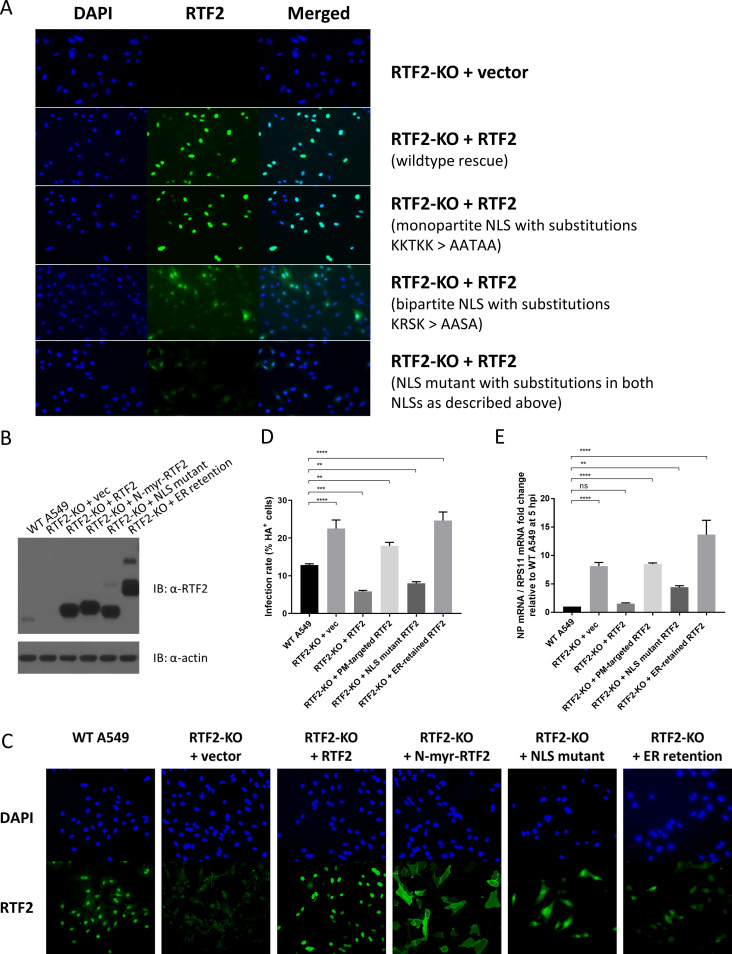
Mislocalization of RTF2 reduces its protective role against IAV. (A) Clonal RTF2-KO cells were transduced with lentiviruses that encode the empty vector, the guide-resistant form of RTF2, or RTF2 with the predicted NLSs mutated. Site-directed mutagenesis was performed to replace lysine or arginine residues with alanine residues. Shown are transduced cells stained with DAPI and anti-RTF2 antibody. RTF2 with intact NLSs localizes to the nucleus. Editing the predicted monopartite NLS had no effect on RTF2’s nuclear localization. However, editing the predicted bipartite NLS seemed to disperse RTF2 throughout the cell. (B) Western blot of WT A549 cells and RTF2-KO cells rescued with either the empty vector or various sgRNA-resistant forms of RTF2: RTF2 (WT RTF2 with intact NLSs), N-terminus-myristoylated (N-myr) RTF2 (RTF2 containing point mutations in both NLSs with an additional plasma membrane-targeting myristoylation signal added to its N terminus), an NLS mutant (RTF2 containing point mutations in both NLSs), or an ER-retention mutant (RTF2 containing point mutations in both NLSs with an additional signal peptide and ER-retaining KDEL motif added). (C) Immunofluorescence of WT A549 cells and clonal RTF2-KO cells transduced with the empty vector or sgRNA-resistant cDNA encoding the various constructs described in the legend to panel B. (D, E) Effect of mislocalizing RTF2 on IAV infection, as assayed by HA staining (D) and qPCR measurement (E). Cells were pretreated with 200-U/ml IFN-β, before IAV infection at an MOI of 5. Flow cytometry, based on cell surface HA, was performed at 16 hpi (D), while qPCR was performed on RNA isolated at 5 hpi (E). *P* values were determined by one-way ANOVA and Dunnett’s multiple-comparison test. ****, *P* ≤ 0.0001; ***, *P* ≤ 0.001; **, *P* ≤ 0.01. PM, plasma membrane.

We then wondered if the nuclear localization of RTF2 is essential for its antiviral function. To do this, we first used the cNLS Mapper program (30) to identify potential classical importin-alpha/beta pathway-specific nuclear localization signals (NLSs) on RTF2. We found two potential NLSs in RTF2: a monopartite NLS (LEKKTKKPKKA) and a bipartite NLS (GATKRSIADSEESEAYKSLFTTHSSAKRSKE). Site-directed mutagenesis of the nucleotide sequences encoding the predicted monopartite NLS (by changing several positively charged residues to alanine residues) had no effect on the location of RTF2, but changing the sequences for the predicted bipartite NLS caused the overexpressed protein to be present in both the cytosol and the nucleus (Fig. 4A). This is perhaps because RTF2 is a small protein (33 kDa) that could still diffuse back into the nucleus upon overexpression despite having both predicted NLSs disrupted.

To test whether RTF2 loses its activity if it is mislocalized such that it cannot diffuse back into the nucleus, we generated an endoplasmic reticulum (ER)-retention mutant (KDEL) and a plasma membrane-targeted N-terminus-myristoylated mutant (Fig. 4B) and showed that the proteins were localized away from the nucleus (Fig. 4C). To test the effect of mislocalizing RTF2 during IAV infection, we quantified infection based on surface HA expression (Fig. 4D) and NP mRNA levels (Fig. 4E). By retaining RTF2 in the endoplasmic reticulum or by redirecting it to the inner leaflet of the plasma membrane, we disrupted RTF2’s ability to confer antiviral protection. On the other hand, point mutations of the NLSs alone had no impact or a weak impact on RTF2 function in the rescued RTF2-KO cells. These data indicate that the RTF2 protein is localized to the nucleus and that relocalization to the plasma membrane or ER impairs its antiviral function.

### Loss of RTF2 leads to increased viral polymerase activity and elevated levels of influenza virus mRNAs synthesized during primary transcription.

**FIG 5:**
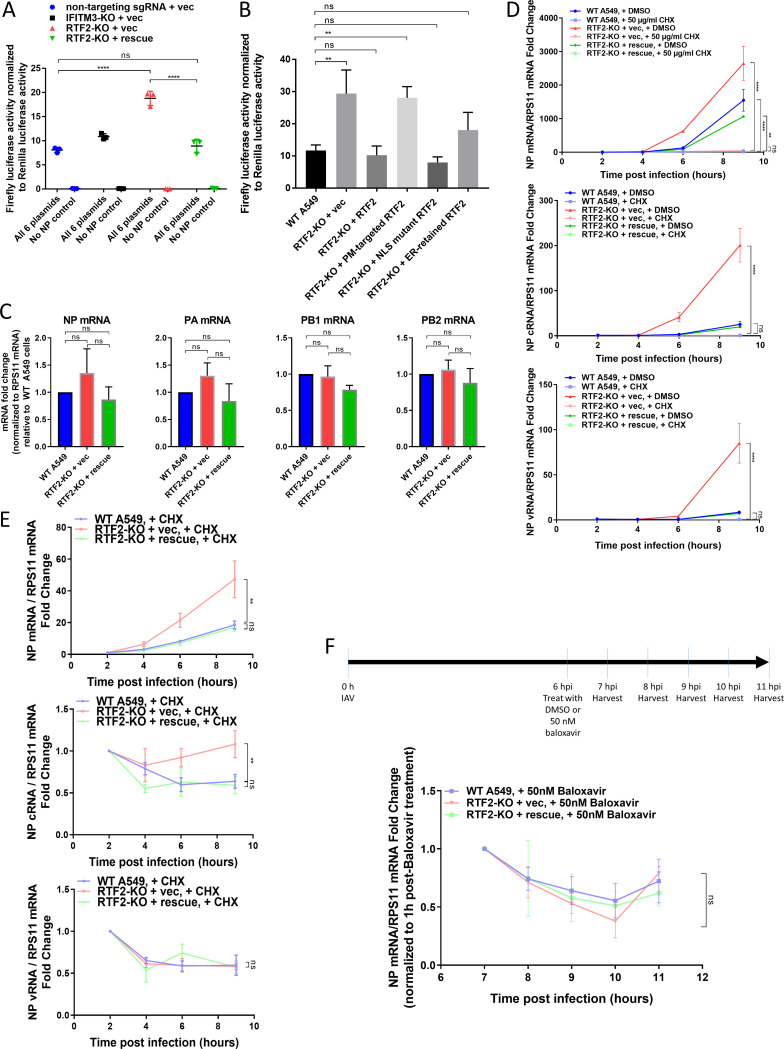
RTF2 restricts a nuclear stage of the influenza virus life cycle and at least blocks primary transcription. (A) IAV polymerase activity was measured via a minigenome luciferase reporter assay. Cells were transfected with plasmids harboring the genes for IAV polymerase subunits and NP, a reverse-orientation firefly luciferase reporter on a vRNA backbone, and a *Renilla* luciferase as a transfection control. Luciferase activity was measured at 24 h posttransfection. ****, *P* ≤ 0.0001, by one-way ANOVA and multiple comparisons. (B) Effect of mislocalizing RTF2 on IAV replication/transcription, as assayed in a minigenome luciferase reporter assay. The cells described in the legend to Fig. 4B were transfected with the plasmids described in panel A. Luciferase activity was measured at 24 h posttransfection. **, *P* ≤ 0.01, by one-way ANOVA and Dunnett’s multiple-comparison test. (C) The transcript levels of transfected plasmids harboring the genes for IAV NP, PA, PB1, and PB2 were quantified in WT A549, RTF2-KO, and RTF2-rescued cells. Cells were transfected for the minigenome luciferase reporter assay as described in the legend to panel B, and RNA was extracted at 24 h posttransfection for qPCR analysis. None of the comparisons reached statistical significance. (D) IAV NP mRNA (top), cRNA (middle), and vRNA (bottom) levels of infected cells were monitored over time in the presence of cycloheximide (CHX) treatment. WT A549, RTF2-KO, and RTF2-rescued cells were pretreated with 200-U/ml IFN-β for 24 h, followed by treatment with either CHX or DMSO for 2 h, and then infected with IAV at an MOI of 5 for 9 h. *P* values were determined by one-way ANOVA and Dunnett’s multiple-comparison test comparing each of the samples collected at 9 hpi against CHX-treated WT A549 cells. ****, *P* ≤ 0.0001; **, *P* ≤ 0.01. (E) IAV NP mRNA (top), cRNA (middle), and vRNA (bottom) levels in IFN-β-pretreated and CHX-treated WT A549, RTF2-KO, and RTF2-rescued cells. **, *P* ≤ 0.01, by one-way ANOVA and Dunnett’s multiple-comparison test comparing each of the samples collected at 9 hpi against CHX-treated WT A549 cells. (F) IAV NP mRNA stability was monitored in infected cells that were treated with baloxavir at 6 hpi. IFN-β-pretreated, IAV-infected cells were treated with baloxavir at 6 hpi to prevent further viral transcription and replication. Total RNA was harvested from infected cells at 1-h intervals from 7 to 11 hpi. One-way ANOVA and Dunnett’s multiple-comparison test were performed separately on samples collected at 10 hpi and 11 hpi, and none of the comparisons reached statistical significance.

Given the requirement of nuclear localization for the antiviral function of RTF2 and since IAV, unlike most RNA viruses, transcribes and replicates its genome in the nucleus, we wondered if RTF2 impacts IAV transcription and/or genome replication. To test this, we transfected wild-type A549, RTF2-KO, and RTF2-rescued cells with a plasmid harboring the gene for the firefly luciferase reporter driven by the viral RNA (vRNA) backbone, along with plasmids harboring the genes for PR8 polymerase subunits (PA, PB1, PB2) and NP (31). We found that RTF2-KO cells had a higher luciferase activity than the other cells, suggesting that cells that lack RTF2 are less able to restrict influenza virus transcription/replication (Fig. 5A).

We then tested whether rescuing RTF2-KO cells with the mislocalized mutants described above would result in luciferase activity lower or higher than that from rescuing cells with the NLS-intact form of RTF2. We found that overexpressing mislocalized RTF2 in the RTF2-KO cells did not reduce the higher luciferase signal seen in RTF2-KO cells relative to that seen in wild-type or RTF2-rescued cells (Fig. 5B), once again indicating that the nuclear localization of RTF2 is important for its function. In addition, we noted that the increased firefly luciferase reporter signal in RTF2-KO cells does not appear to correlate with increased transcription levels of transfected genes for IAV NP, PA, PB1, and PB2 (Fig. 5C), suggesting that the higher luciferase signal is indeed due to increased IAV polymerase activity.

To further dissect RTF2’s role in controlling IAV infection, we tested whether RTF2 specifically affects primary transcription by employing cycloheximide (CHX) to block protein translation. We tracked NP mRNA, cRNA, and vRNA levels after infecting CHX- and IFN-β-pretreated cells with IAV. As CHX treatment blocks the synthesis of new viral proteins and, hence, halts genome replication and secondary transcription, the levels of NP cRNA and vRNA did not increase after infection in CHX-treated cells (Fig. 5D and E). However, in CHX-treated cells, IAV NP mRNA levels (resulting from primary transcription) did go up slightly over time (Fig. 5E). More importantly, NP mRNA levels were higher in RTF2-KO cells than in RTF2-rescued cells, suggesting that RTF2 affects primary transcription. Altogether, these findings are consistent with the model that RTF2-deficient cells allow more primary transcription of the virus.

However, it was unclear whether RTF2 also affects genome replication and/or RNA stability in general. To test the latter, we employed baloxavir, a PA endonuclease inhibitor that blocks cap snatching and, hence, the synthesis of mRNAs. We first infected IFN-β-pretreated cells with IAV for 6 h, before administering baloxavir to prevent the additional synthesis of mRNAs and monitoring the RNA levels over time. We found no evidence that influenza virus mRNA decays at a lower rate in RTF2-KO cells than in RTF2-rescued cells (Fig. 5F), suggesting that the higher mRNA level in RTF2-KO cells might be due to an increased synthesis rate.

In conclusion, RTF2’s localization to the nucleus appears to be important for its function in controlling IAV. While we cannot rule out the possibility that IAV secondary transcription and genome replication are also affected by RTF2, our experiments showed that RTF2-KO cells lead to elevated primary transcript levels at a minimum, likely by affecting mRNA synthesis rather than degradation.

### Cross-talk between RTF2 and the IFN pathway.

**FIG 6:**
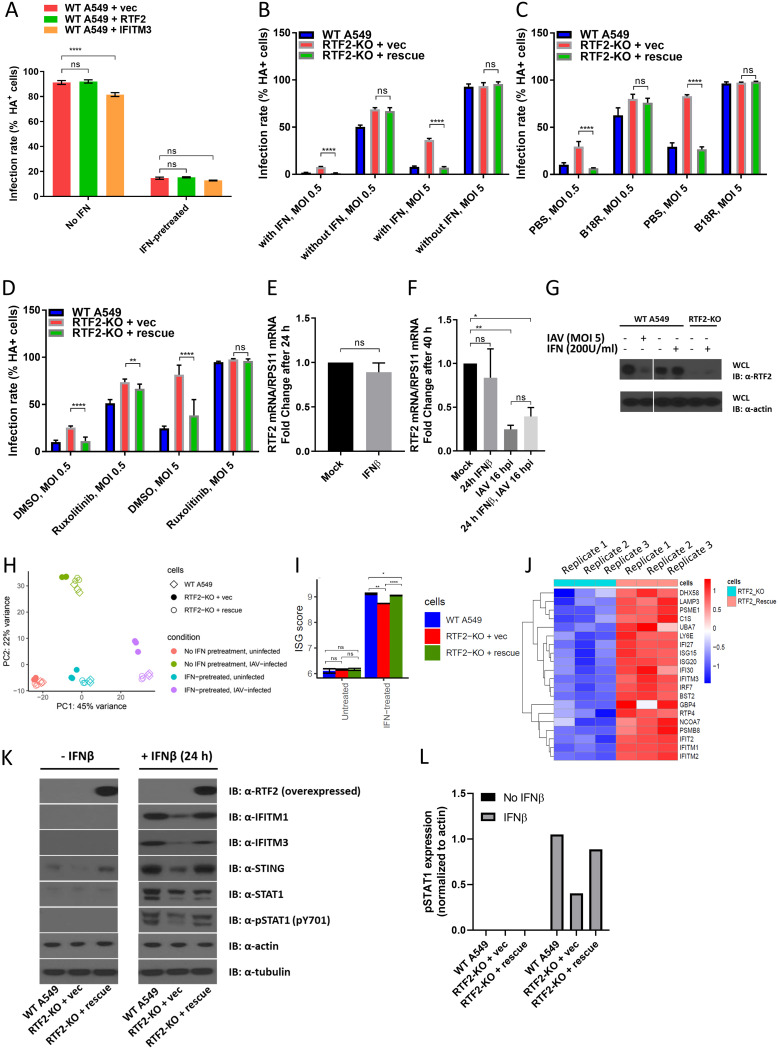
RTF2 may affect the upregulation of antiviral ISGs in response to IFN exposure. (A) Overexpressing RTF2 does not confer additional protection against IAV infection in WT A549 cells. WT A549 cells were transduced with either the empty vector or RTF2. After 8 days of blasticidin selection posttransduction, cells were treated with 200-U/ml human IFN-β for 24 h before IAV infection at an MOI of 5 for 16 h and sorted by FACS based on HA. ****, *P* ≤ 0.0001, by two-way ANOVA and Dunnett’s multiple-comparison test. (B to D) IFN pretreatment enhances the differences in infection rates, as measured by HA staining, between RTF2-KO cells and WT A549/RTF2-rescued cells. (B) Cells pretreated with IFN-β or mock treated. (C) Cells pretreated with IFN-β with or without 0.5-μg/ml B18R, an IFNAR decoy protein. (D) Cells pretreated with IFN-β with or without 5 μM ruxolitinib, a JAK inhibitor. Shown are combined data from two independent experiments with three technical replicates each. *P* values were determined by one-way ANOVA followed by an unpaired *t* test with Bonferroni’s correction between RTF2-KO and RTF2-rescued cells. ****, *P* ≤ 0.0001; **, *P* ≤ 0.01. (E) The RTF2 mRNA level (normalized to the RPS11 mRNA level) appears to be unchanged after 24 h of 200-U/ml IFN-β exposure. (F) Cells were treated with IFN-β (at 200 U/ml for 24 h), infected with IAV (at an MOI of 5 for 16 h), or pretreated with IFN-β for 24 h before IAV infection, before RNA was extracted for qPCR to quantify RTF2 mRNA and RPS11 mRNA levels. *P* values were determined by one-way ANOVA and Bonferroni’s multiple-comparison test. **, *P* ≤ 0.01; *, *P* ≤ 0.05. (G) Western blot showing a reduction of the RTF2 protein level in IAV-infected cells but no such decrease in cells exposed to just IFN-β alone. WCL, whole-cell lysate. (H) Principal-component analysis of gene expression profiles of WT A549, RTF2-KO, and RTF2-rescued cells under different conditions. The top positively weighted genes in PC1 are those for IFITM1, OAS2, and Mx1, and the top positively weighted genes in PC2 are those for NGFR, FOS, and TNFRSF10D. (I) ISG score based on RNA sequencing of WT A549, RTF2-KO, and RTF2-rescued cells with or without IFN (200-U/ml IFN-β for 24 h). ****, *P* ≤ 0.0001; **, *P* ≤ 0.01; *, *P* ≤ 0.05. (J) Comparison of ISGs that are differentially expressed in IFN-β-pretreated RTF2-KO and RTF2-rescued cells. Shown are three technical replicates with the row-normalized values for each gene. (K) Western blot showing phosphorylated STAT1 (pSTAT1; pY701), IFITM1, IFITM3, and STING levels in IFN-β-treated WT A549, RTF2-KO, and RTF2-rescued cells. Cells were mock treated or exposed to 200 U/ml IFN-β for 24 h. (L) Quantification of the pSTAT1 band in panel K.

Since most of our experiments were conducted in the presence of IFN pretreatment, we wondered if IFN is required to observe RTF2’s role in influenza virus restriction. We found no changes in infection when RTF2 was overexpressed (Fig. 6A), indicating that RTF2 alone is not sufficient to restrict viral replication. To investigate the dependence on IFN, we infected wild-type A549, RTF2-KO, and RTF2-rescued cells in the presence or absence of IFN and then measured the infection rates based on surface HA levels. We found that IFN pretreatment (which reduces overall infection rates) enhances the fold increase in infection between RTF2-KO cells and RTF2-rescued cells (Fig. 6B). We adopted two other complementary approaches—by using recombinant B18R (Fig. 6C), a decoy IFNAR protein produced by vaccinia virus to soak up IFN in the culture medium, and by using ruxolitinib (Fig. 6D), a JAK inhibitor—to block IFN signaling. All three approaches suggest that an intact IFN signaling pathway is required to observe the effect of RTF2 knockout (KO) on infection. A potential reason why the RTF2-KO phenotype is observed only in the presence of IFN may be that IFN activates RTF2 or upregulates an unknown cofactor of RTF2. This model remains to be tested. On the other hand, because RTF2 protein and mRNA levels were not upregulated in the presence of IFN (Fig. 6E to G), we hypothesized, alternatively, that RTF2 restricts influenza virus by positively regulating the response to IFN and that the loss of RTF2 would lead to a blunted IFN-induced antiviral response. Interestingly, we also noticed that although RTF2 expression appeared to be unaffected by IFN-β treatment alone, it was significantly reduced upon IAV infection (Fig. 6F and G).

To investigate the hypothesis that RTF2 positively regulates the response to IFN, we performed RNA sequencing to compare the transcriptomes of RTF2-KO and RTF2-rescued cells in the presence of IFN. Principal-component analysis (PCA) showed that one dimension was associated with IFN exposure, while the other dimension was associated with IAV infection (Fig. 6H). While the transcriptomes of RTF2-KO and RTF2-rescued cells were quite similar in the absence of IFN exposure (and IAV infection), RTF2-KO cells showed lower levels of induction of ISGs than wild-type or RTF2-rescued cells (Fig. 6I). Among the reduced ISGs, we found the known influenza virus restriction factors IFITM1 and IFITM3 (Fig. 6J), which validated the findings at the protein level (Fig. 6K). Consistent with this observation, we found that the phosphorylation of STAT1, a major transcription factor responsible for upregulating ISGs during IFN signaling, was reduced in RTF2-KO cells (Fig. 6L), further supporting RTF2’s role in modulating the IFN pathway.

**FIG 7:**
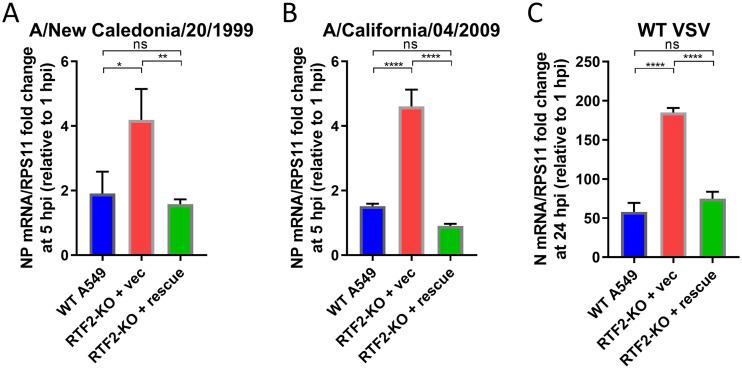
RTF2-KO cells have higher viral RNA levels when infected with other IAV strains and WT VSV. (A, B) WT A549, RTF2-KO, and RTF2-rescued cells were pretreated with 200-U/ml IFN-β before infection with the influenza virus A/New Caledonia/20/1999 (A) or A/California/04/2009 (B) at an MOI of 5 for 5 h. RNA was harvested at 5 hpi for qPCR to measure NP mRNA levels. (C) WT A549, RTF2-KO, and RTF2-rescued cells were pretreated with 200-U/ml IFN-β before infection with WT VSV at an MOI of 5 for 24 h, before RNA was harvested for qPCR to measure N mRNA levels. *P* values were determined by one-way ANOVA and Tukey’s multiple-comparison test. ****, *P* ≤ 0.0001; **, *P* ≤ 0.01; *, *P* ≤ 0.05.

Given that RTF2 may play a role in positively regulating the IFN response, we hypothesized that RTF2 KO should result in higher infection rates by other viruses, especially viruses that are susceptible to the effects of IFN signaling. To test this, we infected RTF2-KO cells with clinical isolates of IAV, such as the 1999 New Caledonia strain and the 2009 California H1N1 pandemic strain, as well as vesicular stomatitis virus (VSV). Consistent with our hypothesis, RTF2-KO cells showed higher NP mRNA levels for both the A/New Caledonia/20/1999 and A/California/04/2009 viruses (Fig. 7A and B) and higher VSV N mRNA levels (Fig. 7C).

Collectively, our data suggest that RTF2 contributes positively to the cellular response to IFN via the IFNAR pathway, as the loss of RTF2 results in lower levels of phosphorylated STAT1 (pSTAT1) and ISGs and impairment of the IFN-induced antiviral response.

## DISCUSSION

In our screen, we used HA expression as a surrogate readout for viral infection and replication. There are inherent caveats to this approach. First, restriction factors that inhibit later stages of the life cycle, such as assembly, budding, and release, as well as subsequent rounds of infection (since we chose to omit trypsin from the cell culture medium), would be missed in our screen. Examples of such antiviral factors that could be missed include viperin and tetherin. In addition, potential host restriction factors that inhibit the expression of non-HA late genes of IAV, such as M2 and neuraminidase (NA), may also be missed. However, any restriction factors that target viral proteins involved in viral gene expression (such as polymerase subunits PA, PB1, and PB2 or an indirect regulator, such as NS1, which inhibits the cellular antiviral response and which traps cellular pre-mRNAs) should be captured in our screen. Any restriction factor that inhibits HA expression and/or that reduces HA stability should also be recoverable in our screen (since knocking out these hypothetical restriction factors would result in higher levels of HA expression). Interestingly, although our screen recovered genes involved in the IFN signaling pathway (the genes for IFNAR1, IFNAR2, JAK1, TYK2, and IRF9), we did not recover previously reported antiviral ISGs. This is in spite of the fact that both the IFITM3 and Mx1 proteins have been reported to be functional in A549 cells. We hypothesized that this might be due to the small effect size of each individual antiviral effector gene, as well as the redundancy of the many ISGs upregulated upon IFN exposure. Hence, knocking out a single ISG may not be sufficient for viruses to overcome the IFN-induced protection. In addition, despite puromycin selection eliminating nontransduced cells, the incomplete deletion of genes by CRISPR would further reduce the small effect sizes of each ISG and make it challenging to observe the enrichment of specific sgRNAs after flow sorting. Interestingly, this observation of not recovering classical ISGs has also been made in another screen recently described by Richardson et al. (19). Similar to our screen, their screen recovered genes in the IFN signaling pathway (such as the genes for IFNAR1, IFNAR2, JAK1, TYK2, and IRF9), as well as IFI6, but not classical ISGs, such as the genes for IFITM3 (4, 15) and TRIM56 (32), which have previously been shown to inhibit yellow fever virus. Potential improvements to such screens in future could involve using a lower IFN dose, using a small validated sgRNA library, increasing the number of cells in the screen to detect smaller effect sizes, and performing a combinatorial screen (e.g., by single-cell approaches to test many combinations) where multiple antiviral genes are knocked out in the same cell.

However, despite not recovering any of the previously reported antiviral ISGs, we found that the loss of RTF2 led to the increased replication of influenza virus, at least in part through an increase in primary transcription and in part through a reduction in the IFN response. This might explain why RTF2 was recovered in our screen when none of the other previously reported antiviral effectors, such as IFITM3, were identified.

While we do not know if RTF2 KO also affects early steps of infection, we found that RTF2-KO cells exhibited a higher rate of primary transcription (rather than a lower rate of degradation). RTF2-KO cells also displayed elevated luciferase activity in the minigenome reporter assay, an assay that is based on transfected plasmids instead of infection with live viruses and, hence, that bypasses all the earlier steps in the viral life cycle. Collectively, these suggest that, at a minimum, RTF2 affects influenza virus polymerase complex activity.

However, we have not resolved whether RTF2’s restriction of influenza virus is due primarily to RTF2 being a direct antiviral effector, a global modulator of the IFN pathway, or both. It is increasingly appreciated that restriction factors could have dual functions (33), with examples such as IFITM3 acting as a feedback inhibitor of type I IFN induction (34), SAMHD1 inhibiting NF-κB and IRF7 activation (35), and viperin enhancing Toll-like receptor signaling and type I IFN production in plasmacytoid dendritic cells (36). However, since RTF2 KO results in lower levels of pSTAT1 and, hence, lower levels of ISG upregulation, the depletion of RTF2 is likely to reduce important direct antiviral effectors (such as IFITM3) and, thus, increase the efficiency of multiple steps in the viral life cycle, including primary transcription. Because we demonstrated increased VSV infection in RTF2-KO cells, we hypothesize that RTF2 would restrict multiple viruses through its regulation of the IFN response.

Although RTF2’s mechanism of action has yet to be worked out, our work advances the field by elucidating a novel cellular function of this understudied gene. Previously, Inagawa et al. reported that the Schizosaccharomyces pombe RTF2 homolog could be involved in site-specific replication termination by stabilizing stalled replication forks at the specific replication barrier *RTS1* element (29), while Sasaki et al. reported that the Arabidopsis thaliana homolog may regulate pre-mRNA splicing in a ubiquitin-dependent fashion to affect embryonic development (37). The only study of RTF2 in human cells was done by Kottemann et al., reporting that RTF2 needs to be removed from stalled replication forks during induced DNA damage in order to maintain genome integrity (28). The one common thread across these studies is that RTF2 appears to affect the processing of nucleic acids. Future studies investigating whether RTF2 interacts with viral RNAs might reveal how RTF2 restricts virus infection, which may be testable in an *in vitro* replication assay with purified components. In addition, identifying RTF2’s interaction partners, perhaps through immunoprecipitation-mass spectrometry, may also potentially shed light on the exact molecular mechanism of RTF2’s antiviral function. Finally, the effect of RTF2 on DNA damage may also impact the IFN response through innate immune pathways triggered by DNA damage (38) and warrants further investigation.

While progress has been made in understanding cellular antiviral defenses, the contributions of the many host antiviral factors to controlling specific viruses have yet to be determined. As each virus evolves a unique strategy to counteract host restriction factors, one may expect to find few pan-virus effectors (15, 18) but, instead, may expect to find many effectors that have directly evolved to cope with different viruses. Elucidation of how RTF2 and other factors restrict viral replication will continue to provide insights into how our cells contend with the multitude of viruses.

## MATERIALS AND METHODS

### Cell culture, reagents, and virus strains.

A549, 293T, and Vero cells (ATCC) were cultured in Dulbecco’s modified Eagle medium (DMEM; Thermo Fisher) supplemented with 10% heat-inactivated fetal bovine serum (FBS; Avantor), 2 mM l-glutamine (Gibco), and 1% penicillin (Corning). The A549-Cas9 cell line was generated by transducing A549 cells with a lentiviral construct (pXPR101) expressing Cas9 and blasticidin deaminase. In order to maintain the heterogeneity of the cells, a polyclonal population of cells of the A549-Cas9 cell line was used for the CRISPR screen and initial validation. For subsequent experiments, a clonal RTF2-KO cell line was used as mentioned above. For IFN signaling inhibition, we used recombinant viral B18R protein (catalog number 8185-BR-025; R&D Systems) and ruxolitinib (catalog number tlrl-rux; Invivogen). Baloxavir was obtained from MedChemExpress (catalog number HY-109025A). Cycloheximide was obtained from Cell Signaling Technology (catalog number 2112). For microscopy, we used rabbit serum for blocking (catalog number R9133; Sigma) and Vectashield antifade mounting medium with DAPI (4′,6-diamidino-2-phenylindole; catalog number H-1200; Vector Labs). The influenza A virus A/Puerto Rico/8/1934 (PR8) was grown in Vero cells in serum-free DMEM supplemented with 1% bovine serum albumin (BSA) and 1-μg/ml tosylsulfonyl phenylalanyl chloromethyl ketone (TPCK)-treated trypsin (Thermo Fisher). TPCK-treated trypsin was obtained from Thermo Fisher (catalog number 20233), and trypsin-neutralizing solution was obtained from Sigma (catalog number 10109886001). Vesicular stomatitis virus (VSV) was kindly gifted by Sean Whelan’s lab. The influenza A viruses A/New Caledonia/20/1999 and A/California/04/2009 were kindly gifted by Daniel Lingwood’s lab.

### Plasmids.

The pXPR101 plasmid used to generate A549-Cas9 cells was provided by the Broad Institute Genetic Perturbation Platform. Individual sgRNAs were cloned into pLentiCRISPR-V2 (Addgene accession number 52961) for follow-up studies. For rescue experiments, the Cas9 gene in pXPR101 was replaced by codon-mutated versions of RTF2. Additional site-directed mutagenesis was performed to generate mislocalized forms of RTF2. For the ER-retention mutant, a signal peptide (MLLSVPLLLGLLGLAAAD) (39) was added to the N terminus, and a KDEL motif was added to the C terminus of the NLS mutant. For the plasma membrane-targeting mutant, a myristoylation motif (GCIKSKRKDNLNDDGVD) (40) was added to the N terminus of the NLS mutant. The plasmids used in the luciferase reporter assay were previously described (41).

### Antibodies.

The following antibodies were used: from EMD Millipore, anti-influenza A virus HA (1:200; catalog number AB1074); from Abcam, anti-IFITM1 (1:2,000; catalog number ab224063) and mouse anti-β-actin antibody (1:15,000; catalog number ab6276); from Thermo Fisher, Alexa Fluor 488-conjugated donkey anti-goat IgG (1:500; catalog number A11055), a rabbit anti-mouse IgG (H+L) secondary antibody Alexa Fluor 488 conjugate (1:500; catalog number A-11059), and mouse anti-alpha-tubulin antibody (1:2,000; clone DM1A; catalog number 14-4502-82); from Cell Signaling Technology, anti-IFITM3 (1:2,000; catalog number 59212T), anti-STING (1:2,000; catalog number 13647), anti-pSTAT1 (1:2,000; clone Y701; catalog number 9167), and anti-TATA-binding protein (1:2,000; catalog number 8515s); and from LifeSpan Biosciences, mouse anti-RTFDC1 antibody (clone 1E8; 1:2,000; catalog number LS-C340588).

### Genome-wide CRISPR screen.

We transduced ∼120 million Cas9-positive A549 cells with the AVANA-4 lentiviral library (21) at a low MOI to achieve a 30% infection rate (such that most cells received only a single sgRNA) and an average of a 500-fold coverage of the library after selection. At 24 h postransduction, cells were subjected to 8 days of puromycin selection to remove nontransduced cells. On day 9 post-lentiviral transduction, ∼300 million cells were subjected to 200-U/ml IFN-β treatment for 24 h and then infected with the influenza A virus PR8 at an MOI of 5 for 16 h. At 16 h postinfection, the cells were washed with PBS and stained with primary anti-influenza A virus HA antibody (catalog number AB1074; EMD Millipore) and secondary Alexa Fluor 488-conjugated anti-goat IgG (catalog number A11055; Thermo Fisher), before fixation with 4% formaldehyde. HA-positive and HA-negative cells were sorted by fluorescence-activated cells sorting (FACS) and harvested for genomic DNA using a Qiagen blood and tissue extraction kit according to the manufacturer’s protocol. PCR of guide DNA (gDNA) was performed in 100-μl reaction mixtures to attach the sequencing adaptors and barcode samples. Each reaction mixture consisted of 50 μl of gDNA plus water, 40 μl of PCR master mix, and 10 μl of a uniquely barcoded P7 primer (stock at a 5 μM concentration). The master mix was comprised of 75 μl *Ex Taq* DNA polymerase (Clontech), 1,000 μl of 10× *Ex Taq* buffer, 800 μl of the deoxynucleoside triphosphates provided with the enzyme, 50 μl of P5 stagger primer mix (stock at a 100 μM concentration), and 2,075 μl water. PCR cycling conditions were an initial 1 min at 95°C, followed by 30 s at 94°C, 30 s at 52.5°C, and 30 s at 72°C for 28 cycles and a final 10-min extension at 72°C. Samples were purified with Agencourt AMPure XP SPRI beads according to the manufacturer’s instructions (catalog number A63880; Beckman Coulter) and sequenced on a HiSeq2000 sequencer (Illumina). The screen results were analyzed with the CRISPR gene scoring tool developed at the Broad Institute, using both the negative binomial distribution (STARS [21] software, version 1.1) and the hypergeometric distribution.

### Virus infection.

A549 cells were inoculated with 150 μl (12-well plate) or 2 ml (T75 flask) of influenza A virus or vesicular stomatitis virus at an MOI of 5 for 1 h at 37°C in serum-free DMEM. The cells were then rinsed once and replaced with fresh serum-free DMEM supplemented with 1% BSA for the length of the respective experiments. Infection was subsequently monitored by FACS or quantitative reverse transcription (RT)-PCR. For experiments involving CHX treatment, cells were exposed to either dimethyl sulfoxide (DMSO) or 50-μg/ml CHX for 2 h toward the end of the 24-h IFN pretreatment, prior to infection; CHX was retained in the infection and cell culture medium until the cells were harvested. For IFN dependence experiments, cells were pretreated with 0.5-μg/ml IFNAR decoy B18R or 5 μM JAK inhibitor ruxolitinib for 2 h prior to IFN-β treatment, and the respective blockers were left in the medium throughout the duration of the subsequent 24-h IFN treatment. For the RNA stability experiment, 50 nM baloxavir was added to the cells at 6 hpi.

### Rescuing RTF2-KO cells.

To rescue RTF2 expression in RTF2-KO cells, an XPR101_rescue plasmid expressing a FLAG-tagged codon-mutated version of RTF2 was used. Cells were selected with 1-μg/μl puromycin and 10-μg/μl blasticidin for 8 days. Expression of rescued RTF2 was confirmed via Western blotting.

### Flow cytometry.

Trypsin was used to lift the adherent cells, before neutralization with DMEM supplemented with 10% FBS. The cells were then rinsed once with cold phosphate-buffered saline (PBS)–1% BSA before being stained with anti-influenza A virus HA (catalog number AB1074; EMD Millipore) in PBS–1% BSA (1:200) for 30 min on ice, rinsed with cold PBS twice, and stained with the secondary antibody, Alexa Fluor 488-conjugated donkey anti-goat IgG (catalog number A11055; Thermo Fisher), at a 1:500 dilution for 30 min on ice. After staining, the cells were rinsed in PBS twice, fixed with 2% formaldehyde, rinsed with PBS, and sorted. Data were acquired on a BD Accuri (BD Bioscience) or CytoFLEX S (Beckman) flow cytometer and analyzed by FlowJo software (TreeStar).

### Plaque assays.

A549 cells, seeded on 12-well plates, were pretreated with 200 U/ml of IFN-β for 24 h, before they were infected with influenza A virus at an MOI of 5 for 1 h at 37°C in serum-free DMEM. After 1 h of inoculation, the cells were rinsed once with serum-free DMEM, before they were left in 1 ml of serum-free DMEM supplemented with 1% BSA for another 15 h. At 16 h postinfection, the supernatant was collected and subjected to activation with TPCK-treated trypsin (final concentration, 16 μg/ml) for 1 h at 25°C, while the A549 cells were harvested for FACS analysis. After 1 h of trypsin activation, trypsin inhibitor was added to a final concentration of 16 μg/ml to fully neutralize the trypsin, before 10-fold serial dilutions were made in serum-free DMEM supplemented with 1% BSA. The diluted viruses (250 μl) were added to a confluent monolayer of Vero cells on 6-well plates (3.6 × 10^5^ cells/well seeded on the day before) for the plaque assay. After 1 h at 37°C, the inoculum was aspirated before a 0.25% agarose overlay (1× minimal essential medium, 0.35% BSA, 0.12% [wt/vol] NaHCO_3_, 1× penicillin/streptomycin, 2 mM glutamine, 25 mM HEPES, 0.25% agarose, 1 μg/ml TPCK-treated trypsin) was cast over the infected Vero cells. After the agarose overlay solidified, the cells were then incubated at 37°C for 48 h, before they were fixed with 10% (vol/vol) formaldehyde in 1× PBS and stained with 0.05% (wt/vol) crystal violet in 10% ethanol to visualize the plaques. The virus titer was then calculated and is given as the number of PFU per milliliter.

### Western blotting.

Transduced cells (5 × 10^5^ cells) were lysed, after the different treatment conditions stated in the relevant experiments, in radioimmunoprecipitation assay (RIPA) buffer (Thermo Fisher) supplemented with EDTA-free protease inhibitor cocktail (Roche), unless otherwise stated. Cell lysates were centrifuged at 10,000 × *g* in a microcentrifuge for 10 min at 4°C to clear the debris. SDS loading buffer and dithiothreitol were added to the collected supernatant before heating at 95°C for 5 min to denature the proteins. Gel electrophoresis was performed by separating the proteins on a NuPAGE Novex 12% Tris-glycine gel, and the proteins were transferred to a polyvinylidene difluoride membrane (Millipore). Immunoblotting was performed according to standard protocols using the relevant primary antibodies (listed under “Antibodies” above) and horseradish peroxidase-conjugated secondary antibodies.

### RNA-extraction and qPCR.

Total RNA was extracted from 1 × 10^5^ cells using an RNeasy minikit (Qiagen) according to the manufacturer’s protocol. First-strand cDNA synthesis was performed using 50 ng of total RNA with a SuperScript III first-strand synthesis system with oligo(dT) (Thermo Fisher). qPCR was performed using a Q5 hot-start High-Fidelity polymerase and SYBR green I nucleic acid gel stain (Thermo Fisher) on a Roche 480 light cycler (Roche). Human RPS11 was used as a reference normalization control, and expression levels were quantified by the delta delta threshold cycle method. Primer sequences were as follows: for influenza virus PR8 NP (42), forward primer 5′-ACCAATCAACAGAGGGCATC-3′ and reverse primer 5′-TGATTTCGGTCCTCATGTCA-3′; for influenza virus PR8 PA, forward primer 5′-GCAAAGTCGGTATTCAACAG-3′ and reverse primer 5′-GGGATCATTAATCAGGCACT-3′; for influenza virus PR8 PB1, forward primer 5′-TAAGCACTGTATTAGGCGTC-3′ and reverse primer 5′-CTTCATGATTGGGTGCATTC-3′; for influenza virus PR8 PB2, forward primer 5′-CGGATCATCAGTCAAGAGAG-3′ and reverse primer 5′-ATCAATCTCCTGGTTGCTTT-3′; for influenza virus A/New Caledonia/20/1999 NP, forward primer 5′-TGAGGGACGACTGATCCAGA-3′ and reverse primer 5′-ATGTGAGTCAAACCAGCCGT-3′; for influenza virus A/California/04/2009 NP, forward primer 5′-GCTTGTGTGTATGGGCTTGC-3′ and reverse primer 5′-TCTGGACCCCTCTTGTGGAA-3′; for RPS11, forward primer 5′-TACCAAAAGCAGCCGACCAT-3′ and reverse primer 5′-CCCTCAATAGCCTCCTTGGG-3′; and for VSV N, forward primer 5′-ATCGGGAAAGCAGGGGATAC-3′ and reverse primer 5′-TTTGTCATCTGCGCTGGTTC-3′. In the RT for influenza virus PR8 NP, the gene- and sense-specific primers (42) were 5′-AGTAGAAACAAGGGTATTTTTC-3′ for NP cRNA and 5′-AGCAAAAGCAGGGTAGATAATCACTCAC-3′ for NP vRNA.

### RNA sequencing.

The Smart-Seq2 protocol (43) was employed to perform transcriptomic analyses of the different cells. Total RNA was extracted using an RNeasy minikit (Qiagen) according to the manufacturer’s protocol. The cDNA was synthesized from 1 ng of total RNA using SuperScript II reverse transcription, followed by PCR amplification and a quality check using a high-sensitivity DNA Bioanalyzer chip (Agilent). cDNA (0.15 ng) was then used for the tagmentation reaction, carried out with a Nextera XT DNA sample preparation kit (Illumina), and further PCR amplification. Paired-end reads of 38 bp of the amplified library were generated on a NextSeq 500 instrument (Illumina) and aligned to the hg19 (GENCODE [v21] software) transcriptome using STAR (v2.6) software. RSEM (v1.3.1) software was then used to quantify the expression of all genes. The RNA sequencing data have been deposited in the NCBI Gene Expression Omnibus (GEO) database. Downstream analysis of the resulting gene × sample expression matrix was performed in R software. Specifically, differential expression was calculated with the R package DESeq2. The IFN response score was calculated by summing the DESeq2 normalized expression values of the ISGs obtained from the hallmark interferon alpha response gene set.

### Microscopy.

Cells were grown in chamber slides (Nunc Lab-Tek II chamber slide system; Thermo Fisher) overnight. The medium was aspirated, and then the cells were rinsed with 1× PBS, before they were fixed in fresh 4% formaldehyde for 15 min at room temperature. The cells were then rinsed with 1× PBS thrice for 5 min each time, before they were permeabilized and blocked in rabbit serum for 1 h at room temperature and then stained with anti-RTF2 primary antibody (1:250; catalog number LS-C340588; LifeSpan Biosciences) at 4°C overnight. The cells were then rinsed with 1× PBS thrice for 5 min each time and then stained with rabbit anti-mouse immunoglobulin secondary antibody conjugated with Alexa Fluor 488 (catalog number A-11059; Thermo Fisher) for 1 h at room temperature in the dark. The cells were then rinsed with 1× PBS thrice for 5 min each time and then mounted and allowed to cure overnight before imaging. For live-cell imaging, mCherry-tagged RTF2-expressing cells were grown in regular 6-well plates in DMEM. Cells were imaged on a wide-field epifluorescence microscope (Applied Scientific Instrumentation), with the images acquired using Micro-Manager software. The acquisition settings and exposure times were kept consistent across experiments.

### Luciferase reporter assay for influenza A virus replication.

To measure viral polymerase activity, we utilized a vRNA-luciferase reporter system. Briefly, A549 cells were transfected with a vRNA reporter plasmid expressing firefly luciferase under a viral untranslated region. The cells were also transfected with influenza A virus PA, PB1, PB2, and NP and *Renilla* luciferase plasmids. At 24 h posttransfection, the cells were lysed and mixed with Dual Glo substrate (Promega) according to the manufacturer’s protocol. Luminescence was measured and quantified using a Synergy H1 multimode microplate reader (BioTek).

### Biochemical fractionation.

The cell fractionation protocol was adapted from the Ficoll-digitonin protocol previously described (44). Briefly, 1.5 × 10^6^ trypsinized WT A549 cells were washed with PBS twice and resuspended in 600 μl of HEPES-sucrose-Ficoll (HSF) solution (20 mM HEPES-KOH, 6.25% Ficoll, 0.27 M sucrose, 3 mM CaCl_2_, 2 mM MgCl_2_) with 50-μg/ml digitonin and EDTA-free protease inhibitor cocktail (Roche). The cells were kept on ice for 10 min, before they were spun down at 1,000 × *g* for 3 min. The supernatant was collected and then further centrifuged at 15,000 × *g* for 10 min to generate the cytosol fraction. The nucleus pellet from the first centrifugation was rinsed with HSF buffer once and spun down again at 1,000 × *g* for 3 min. The supernatant was collected as the washed fraction, and the pellet was then lysed in 50 μl of RIPA buffer with EDTA-free protease inhibitor cocktail (Roche) on ice for 20 min. The lysed nuclei were then centrifuged at 10,000 × *g* for 10 min, before the supernatant was collected as the nuclear fraction for Western blot analysis. The antibodies used for this experiment included mouse anti-alpha-tubulin antibody (clone DM1A; catalog number 14-4502-82; Thermo Fisher), anti-TATA-binding protein (catalog number 8515s; Cell Signaling Technology), and mouse anti-RTFDC1 antibody (clone 1E8; catalog number LS-C340588; LifeSpan Biosciences).

### Statistics and schematics.

Data were tested for statistical significance with GraphPad Prism software. A *t* test, one-way analysis of variance (ANOVA), and two-way ANOVA were performed with their respective multiple comparisons, as indicated in the figure legends. All data are represented as the mean ± standard deviation. The images in the schematics were created using BioRender software.

### Data availability.

The RNA sequencing data are available in the Gene Expression Omnibus (GEO) database under accession number GSE146403.
